# Large-Scale Conformational Changes and Protein Function: Breaking the *in silico* Barrier

**DOI:** 10.3389/fmolb.2019.00117

**Published:** 2019-11-05

**Authors:** Laura Orellana

**Affiliations:** ^1^Institutionen för Biokemi och Biofysik, Stockholms Universitet, Stockholm, Sweden; ^2^Science for Life Laboratory, Solna, Sweden

**Keywords:** conformational change, proteins, molecular dynamics simulation, coarse-grained (CG) methods, structural ensemble

## Abstract

Large-scale conformational changes are essential to link protein structures with their function at the cell and organism scale, but have been elusive both experimentally and computationally. Over the past few years developments in cryo-electron microscopy and crystallography techniques have started to reveal multiple snapshots of increasingly large and flexible systems, deemed impossible only short time ago. As structural information accumulates, theoretical methods become central to understand how different conformers interconvert to mediate biological function. Here we briefly survey current *in silico* methods to tackle large conformational changes, reviewing recent examples of cross-validation of experiments and computational predictions, which show how the integration of different scale simulations with biological information is already starting to break the barriers between the *in silico, in vitro*, and *in vivo* worlds, shedding new light onto complex biological problems inaccessible so far.

## Conformational Changes: Linking Shape and Function

**Figure 1:**
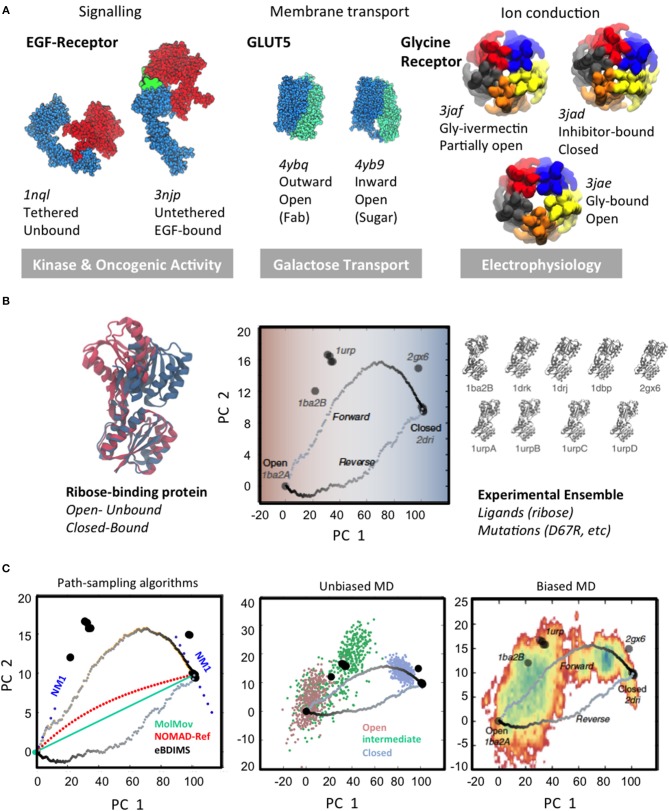
Large-scale conformational changes and different scale sampling methods. **(A)** Three examples of transitions of different scales linked to biological function: left, large-scale domain rearrangement in EGFR upon ligand binding; center, rearrangement of tandem repeats in sugar porters; right, cooperative pentamer motions in pentameric ligand-gated ion channels. The majority of conformations trapped by structural techniques correspond to the extreme, lowest-energy states of biological cycles. **(B)** Experimental conformational landscapes for the hinge-bending transition of the Ribose Binding Protein (RBP) as computed from Principal Component Analysis: the open to closed RBP conformational change upon ribose binding (*Left*); RBP conformational landscape and eBDIMS coarse-grained (CG) transitions (*Center*) as projected onto the PCs derived from the 9 solved structures (*Right*). Note how eBDIMS paths approach the sequence of experimental intermediates. **(C)** Comparison of sampling strategies: NMs and path-finding CG-methods (*Left*); atomistic MD unbiased (500 ns from each unbound state) (*Center*) and 1 μs-biasing to the closed state (*Right*). Note how the first NM derived from both RBP end-states (*Left*) points to the experimental intermediates; note also how eBDIMS paths (gray) roughly follow the MD/X-ray sampled area. Adapted by the author with CC BY license from Orellana et al. (2016).

Protein structure and dynamics are essential to understand living organisms at the molecular level. Already 60 years ago Feynman envisioned that life is, roughly speaking, not only about atomic organization, but also about the “*jiggling and wiggling of atoms”* (Feynman et al., 1963). The central paradigm of structural biology stated that the 3D-fold of a protein is encoded in the sequence (Dill and Chan, 1997; Wright and Dyson, 2015); the explosion of structural data in the past decades has dramatically expanded this classical view, confirming Feynman's prediction. Far from being static structures, it is now clear that proteins rather behave as *living* entities (Henzler-Wildman and Kern, 2007), ever-changing on temporal and spatial scales spanning several orders of magnitude: from local loop fluctuations in enzyme active sites (Aglietti et al., 2013; Pal et al., 2016) to concerted beta-sheets motions (Fenwick et al., 2014) or large-scale allosteric motions in transmembrane receptors (Bugge et al., 2016). Importantly, growing evidence indicates that these large conformational changes are intrinsically encoded in the overall 3D-shape (Bahar et al., 2010), and that external stimuli –binding, post-translational modifications, electrochemical gradients, etc.—just drive these “natural” motions further to trigger output responses. Signal transduction, membrane transport or synaptic communication, almost every cell process relies on switches that cycle between distinct states to allow for bioregulation (Figure 1A). The way that proteins change to sense and respond to such stimuli is therefore central to connect the micro-, meso-, and macro-scales in biology. However, their elucidation from atomic “*jiggling and wiggling”* is far from trivial.

During the past decade, structural determination techniques have made incredible progresses in resolving structures of increasing complexity and flexibility. Currently, high-throughput time-resolved X-ray crystallography (Levantino et al., 2015; Neutze et al., 2015; Ourmazd, 2019), cryo-Electron Microscopy (cryo-EM; Nogales and Scheres, 2015; Murata and Wolf, 2018; Shoemaker and Ando, 2018), and Nuclear Magnetic Resonance (NMR; Baker and Baldus, 2014; Jiang and Kalodimos, 2017; Opella and Marassi, 2017), together with complimentary techniques like Small Angle X-ray Scattering (SAXS; Vestergaard, 2016), Förster Resonance Energy Transfer (FRET; Okamoto and Sako, 2017), double electron-electron resonance (DEER; Jeschke, 2012), mass spectrometry (Kahsai et al., 2014) or fluorescence microscopy (Lewis and Lu, 2019) are allowing to resolve and gain dynamic information from extremely challenging systems. Despite such advances, the experimental study of protein transitions is still demanding. A complete understanding of equilibrium dynamics requires sampling both the structure space available and the underlying free-energy landscape (FEL; Frauenfelder et al., 1991; Zhuravlev and Papoian, 2010a; Nussinov and Wolynes, 2014; Röder et al., 2019) along its relevant dimensions (Figures 1B,C). Ideally, a completely rational and quantitative FEL characterization should stem from first principles, for example, using methods like Molecular Dynamics (MD; Karplus and McCammon, 2002; Orozco, 2014), in which Newton's equations are integrated over time for an atomistic model of the system based on physical potentials. In practice, atomistic-level sampling of the functional FEL of biomolecules poses by itself a huge conceptual and technical problem *in silico*. Collective rearrangements and allosteric events in proteins can involve scales around *ms-*μ*s* and up to 10^2^Å. Note that this is far beyond what classical MD can address in terms of time and size: roughly two orders of magnitude larger than average simulated interatomic distances (~1–10Å), and up to 9–12 orders of magnitude larger than the smallest simulated timestep (fs oscillations) (Sweet et al., 2013). Importantly, functional transitions often occur in this blurred frontier between theory and experimentation.

Scalable codes, graphic processing units (GPUs), parallelization and optimized simulation algorithms (Pierce et al., 2012; Sweet et al., 2013; Kutzner et al., 2015; Páll et al., 2015; Pouya et al., 2017) are however making increasingly feasible to simulate systems with millions of atoms for few μ*s*, or even whole bacterial cytoplasms in the submicrosecond range (Yu et al., 2016). Still, for most proteins, these timescales cover a small part of the structural landscape, and longer simulations are only accessible with special-purpose supercomputers like Anton (Shaw et al., 2009; Dror et al., 2012). Apart from these technical aspects, there is a fundamental “sampling problem,” not efficiently addressed by long simulations: transition paths in a multidimensional landscape are intrinsically stochastic—there are multiple possible transition routes, subject to random fluctuations that unpredictably push over energy barriers. Multiple evidences indicate that the way in which the configuration space is sampled is thus more critical than simulation length. For example, while in μ*s*-long MD, full transitions are still rarely observed, in certain conditions e.g., upon relaxation after removing ligands (Nury et al., 2010; Calimet et al., 2013; Degiacomi, 2019) or introducing mutations (Smolin and Robia, 2015; Orellana et al., 2019b) they can occur in significantly shorter times. Similarly, coarse-grained (CG) methods like Elastic Network Models (ENMs; Mahajan and Sanejouand, 2015), are also capable to predict with striking accuracy, just from the overall shape of a protein, not only the conformational changes observed experimentally but also entire sequences of on-pathway intermediates (Orellana et al., 2016). This suggests that large-scale motions like those defining protein functional FELs may be better understood as collective, supra-atomistic and higher-scale phenomena. Whatever the theoretical framework chosen to explore this issue, the validation of *in-silico* predicted mechanisms is becoming a central question, as quantitative analysis become essential to rationalize the growing dynamical information from techniques like cryo-EM (Frank, 2018; Bonomi and Vendruscolo, 2019).

Let's now imagine the reader wants to know how a series of conformations for a given protein are related, to get insight into some biological mechanism. It is appropriate then to ask: *Can in silico methods really predict conformational transitions? Have such in-silico transitions been validated and how?* This review is intended to provide the non-specialist with some answers to these questions, first raised by Weiss and Levitt (2009). On the first part (Table 1), we will briefly review theoretical methods to predict transition pathways, focusing on the two most common approaches to explore the FEL between two states: either increase atomistic MD sampling (Maximova et al., 2016) or coarse-grain the model of the system (Zheng and Wen, 2017). On the second part (Table 2), we will discuss recent examples from our group and others attempting cross-validation between theory and experiments in this context. This review does not aim to provide an in-depth description of specific methods which can be found elsewhere (Bernardi et al., 2015; Maximova et al., 2016; Mori et al., 2016; Zheng and Wen, 2017; Harpole and Delemotte, 2018). We rather intend to provide general readers, and specially experimentalists, with a broad overview of the most accessible approaches to explore a transition for a typical protein, along with possible validation strategies. Our goal is to help the reader grasp the current potential of *in silico* methods to explain biological phenomena from microscopic scales, and the exciting boundaries we are reaching.

**Table 1:** Summary of common *in silico* methods to explore protein conformational changes (^*^CV-based, ^**^only for setup/short run).

**Goal**	**Methodology**	**Approach**	**Variants**	**Examples**	**Web server**
Transition Ensembles	Molecular Dynamics	Conventional MD Long timescale MD	– Special computer architectures Specialized algorithms System coarse-graining	– Anton GPUs Long-timestep MARTINI simulations	http://www.charmm-gui.org/?doc=input ** http://mmb.irbbarcelona.org/MDWeb/ ** – – – http://molsim.sci.univr.it/mangesh/index.php ** http://cgmartini.nl/index.php/322-charmm **
		Enhanced sampling	Multi-replicate methods	Replica-exchange Weighted ensemble	– –
			Directed sampling	Essential dynamics Dynamic importance sampling* Adaptive sampling (MSM and others)*	– – –
			FEL modification	Accelerated MD (aMD) Umbrella sampling (US)* Metadynamics*, MSM-MTD	– – —
Path-generation	Geometric morphing	Stereochemical restraints	Linear interpolation Rigid-body interpolation Geometric targeting Robot motion planning	MolMovDB FATCAT FRODA Probabilistic roadmap algorithms, etc.	http://www2.molmovdb.org/ http://fatcat.godziklab.org/
	MΔ-path finding	Step-wise generation of transition path	Perturbation methods Chain-of-states	Steered MD*, Targeted MD* String method*, Nudged elastic band*	– –
	CG-path finding	Simplified protein representation	Iterative NMA Simulations (MC, BD)	iMODS NMSIMs CABS-flex dMD eBDIMS	http://www.charmm-gui.org http://molsim.sci.univ.it/mangesh http://biocomp.chem.uw.edu.pl/CABSflex2/index http://mmb.irbbarcelona.org/GOdMD/ https://ebdims.biophysics.se/
	Hybrid methods		Pulling and minimization	Climber	–

**Table 2:** Examples of cross-validation of *in silico-*predicted properties with experiments to specifically probe conformational changes.

**System**	**Simulation technique**	**MD observation and hypotheses**	**Observables**	**Experimental validation**	**References**
Heterotrimeric Gα-GDP	μs-Long MD Mutant simulations	Spontaneous opening/closing of Gα-GDP in absence of GPCR Domain separation disrupts the GDP-site facilitating nucleotide release	Interdomain distances Nucleotide-exchange rates	DEER spectroscopy confirms multiple peaks for inter domain distance distributions with spin labels Fluorescence GTP-binding kinetics of a G-protein tether construct that restricts domain separation slows down nucleotide exchange	Dror et al., 2015
EAAT	Essential dynamics MD	Substrate transport intermediate forms the anion-selective conduction pathway	Anion currents	Trp-scanning mutagenesis and fluorescence quenching of predicted pore-forming residues confirms their interactions with anions Single channel conductance and anion selectivity of mutations of pore-lining residues	Machtens et al., 2015
Importin	sub-μs MD	Spontaneous transition toward extended conformations in water, and compaction in apolar environment	Intramolecular distances	FRET of a dual-fluorophore labeled importin confirms contraction in hydrophobic environment	Halder et al., 2015
SemiSWEET	μs-Long MD	Spontaneous transition from outward-open to inward-open state, through an occluded intermediate	3D-structure of previously unobserved inward-open state Transport activity	Crystallographic validation with structure of a mutant in the inward-open state Alanine mutagenesis of key residues in the extra- and intra-cellular gates and the sugar binding pocket	Latorraca et al., 2017
Arrestin	μs-Long MD	Motions at the two GPCR-binding interfaces (gate-loop and C-loop) are allosterically coupled via interdomain twisting	Separation between labels at the binding interfaces	Mutagenesis Fluorescence spectroscopy	Latorraca et al., 2018
GLIC	μs-Long MD Mutant simulations	Potentiation in Propofol-sensitive mutations is caused by conformational changes expanding transmembrane binding sites	Ion currents	Electrophysiology with voltage-clamp Mutagenesis	Heusser et al., 2018
Enzymatic micromotors	Accelerated MD	Flexibility near the active site mediates catalysis and coupled motion	Enzymatic activity Motor activity	Increased enzyme rigidity upon inhibitor binding reduces catalytic rates and motor speed	Arqué et al., 2019
PTEN	Multirun ns-MD	Conformational change upon phosphorylation that facilitates binding to Ki-67	Protein-protein interaction	Mutation of the predicted interacting sequences abrogates binding and biological effects	Ma et al., 2019
EGFR	μs-Long MD	Local intrinsic disorder of the EGFR kinase Higher dimerization and phosphorylation activity of L834R mutant	Local disorder Dimerization	H/D exchange measurements Light scattering + BN-PAGE	Shan et al., 2012
	μs-Long MD	Hinge-bending motions and overall position in the membrane affected by glycosylation	Epitope accessibility	Antibody C225 binds independent of glycosylation Antibody 2E9 binds preferentially glycosylated EGFR	(Kaszuba et al., 2015)
	ENMs Mutant simulations	*Primary observation:* Ectomutations untether the receptor and displace a domain from a cancer-specific cryptoepitope *Predictions:* Epitope-806 is coupled to the kinase Convergence of missense ectomutations and ectodeletions to activate the kinase Oncogenic activity of untested mutations	Conformational shifts Epitope accessibility Kinase activity Tumor growth rates Therapeutic response Protein design	SAXS dynamic equilibrium between tethered and untethered conformers shifted by mutations FACS binding to mAb806 increased in ectomutations mAb806 binding increases and decreases depending on kinase configuration *In silico*-designed double mutant has synergistic effects *in vitro* (FACS, SAXS) and *in vivo* Ectomutations and ectodeletions respond to mAb806 therapy in animal models	(Orellana et al., 2014, 2019b)

## From Static Snapshots to Multi-State Structural Ensembles

Since the first structure was determined by X-ray crystallography in the late 50s (Kendrew et al., 1958), the number of protein structures deposited every year in the Protein Data Bank (Berman et al., 2000) has been growing exponentially, from a few dozens in the 90s up to over 10,000 structures/year in the past 2 years. As of 2019, we know around 140 thousand native-like protein structures, with resolutions as low as 0.5Å. For a majority of them however, the conformers solved represent the equilibrium end-structures along their functional cycles, typically composed of at least two different meta-stable states (Figure 1A): active/inactive, bound/unbound, open/closed, etc. For such average proteins (Figure 1B), the native apo state frequently populates the deepest basin and spontaneously samples another of comparable or reduced depth, favored by stimuli like binding, post-translational modifications, etc. that shift the population (Nussinov and Wolynes, 2014). Structural determination techniques usually trap conformations near one of such low-energy basins, while the short-lived intermediates connecting them—which can be key to grasp mechanisms (see e.g., Machtens et al., 2015; Orellana et al., 2019b)—are often elusive both experimental and computationally.

To explore the conformational space, structures are typically solved in multiple conditions e.g., introducing mutations, modulating pH, ions, or complexing with molecules—from ligands to antibodies, affibodies, or small drugs. This contributes to enormous redundancy in the PDB, but at the same time, it is a powerful approach to catch intermediates along transitions. For a growing number of intensely studied proteins the multitude of conditions that has been used to determine their 3D-structures has gradually covered the entire conformational landscape. Especially cryo-EM is allowing to routinely obtain protein snapshots in multiple states with each data deposition [see e.g., the Glycine Receptor (GlyR; Du et al., 2015) in Figure 1A] and, although limited to a few protein families, this is revealing the first glimpses into structural ensembles that cover nearly-complete conformational landscapes (Frank, 2018; Bonomi and Vendruscolo, 2019; Hofmann et al., 2019).

**Figure 2:**
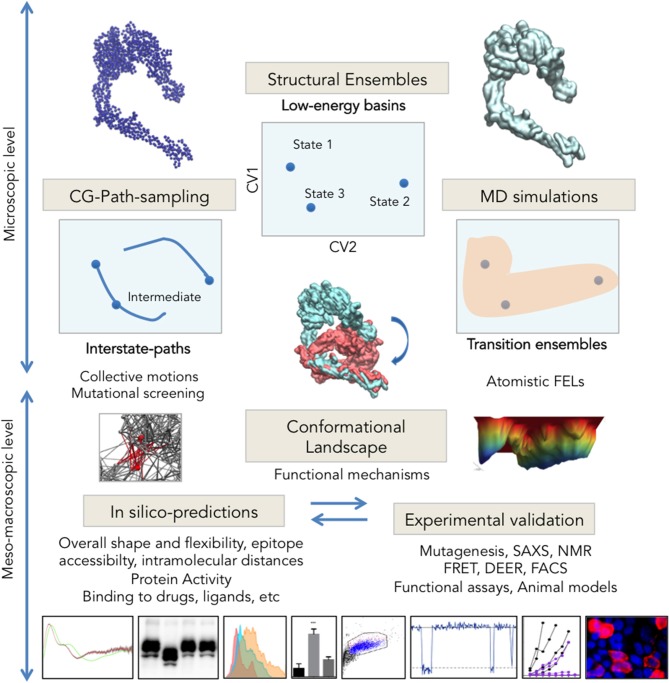
Integrative multi-scale structural biology. Experimental techniques are solving an increasing number of structures trapped in different energy basins, which through ensemble-analysis like PCA can yield intrinsic CVs for landscape exploration **(Top Center)**. Transition pathways computed e.g., from CG-models **(Right)** connect experimental states allowing for intermediate assignment, provide insights into collective motions and can facilitate identification of key regions for mutational analysis. Long or enhanced MD simulations sample the configuration space with atomistic detail and allow reconstructing the complete FEL. Integration of microscopic data on conformational changes generates higher-scale predictions on protein shape, activity and interactions **(Bottom)** that can be tested through structural and molecular biology (microscopy techniques, SAXS, functional assays, etc. See examples in Table 2).

Obtaining multiple snapshots of a protein is however just the first step to characterize its transitions. The second consists on understanding their relationships, which also implies identifying the relevant collective variables (CVs; Kitao and Go, 1999; Zhuravlev and Papoian, 2010b; Noé and Clementi, 2017) for each system. This task is comparable to taking multiple pictures of a moving animal in diverse situations, and then trying to reconstruct its biomechanics; one needs first to find a way to measure, classify and organize the images, so that an ordered sequence can be reconstructed. How are we going to efficiently describe the system? What are we going to measure to detect changes from one functional state to another? Fortunately, large-scale transitions can be often described by a remarkably low number of CVs (Henzler-Wildman and Kern, 2007). This is not surprising since, for most proteins, functional movements are collective: each level of protein motion translates into the next, creating wider and slower movements. For example, local atomic vibrations are transmitted via hydrogen bond networks that make up secondary structures, creating higher amplitude motions; as shown in Fenwick et al. (2011, 2014) the coupled movements of interacting atoms in beta-sheet motifs create collective bending and twisting motions, which propagate to higher collective movements linked to allosteric regulation. Another recurring motif in large-scale protein transitions are open-to-closed motions upon binding (Flores et al., 2006; Amemiya et al., 2011), which in their simplest version consist in rigid-body displacements around a cracking hinge (Figure 1A, left). The hinge region, often located near a binding pocket, is typically an interdomain linker; in more complex transitions wider intra/inter-molecular surfaces can reshape as hinges e.g., in the “rocker-switch” motions between tandem repeats of solute transporters (Drew and Boudker, 2016; Figure 1A, center). Linker or interface reshaping propagate across structures triggering large-scale rearrangements. Usually, such rigid-body transitions are tracked with *ad-hoc* defined angles, distances, etc. However, while for simple hinge-bending transitions, an angle defined by moving rigid bodies can render a fair description of the process, the situation changes when systems undergo complex concerted changes: to accurately describe e.g., gating for ion channels like GlyR (Figure 1A, right) typically demands multiple variables describing extra- and intra-cellular motion features, much harder to define. In such cases, if the protein in question has solved structures in different basins, Principal Component Analysis (PCA; Jolliffe, 2002; Abdi and Williams, 2010), can provide a “natural” representation of the conformational landscape (Sankar et al., 2015) in the form of experimentally-encoded CVs. Compared to other approaches for semi-automated conformer annotation (e.g., based on machine learning; Ung et al., 2018), PCA does not need a priori system-tailored structural descriptors, requiring minimal user intervention PCA. PC-projections approach was recently applied in spliceosome cryo-EM to perform conformer classification, understand its dynamics and obtain a fist assessment of the FEL straight from experimental data (Haselbach et al., 2017, 2018). Moreover, PCs from multi-state ensembles behave as intrinsic complex coordinates that “contain” the heuristic CVs typically defined for each system. As we will discuss later, when such ensemble-analysis are combined with path-sampling, they can illuminate relationships between multiple basins and accurately assign intermediate states, allowing reconstruction of the landscape and its transitions into its experimentally-defined CVs (Figure 2). For example, the Ca^2+^ pump SERCA, with over 70 structures and at least four different states along its complex pumping cycle, constitutes an exceptional example of a multi-basin ensemble where such analysis is critical to unambiguously assign and order experimental on-pathway intermediates (see Orellana et al., 2016). Importantly, PCA of such “*structurally-rich*” or multi-state ensembles provides a much needed and stringent test for any modeling technique to explore protein FELs. In the next two sections, we will review the two most popular and accessible approaches to perform such *in silico* exploration to “connect” experimental basins and “fill in” the conformational landscape: first, sampling with classical MD and its many derivatives, and second, path-finding with computationally simpler methods.

## Exploring the Landscape: Sampling Long vs. Tricked

MD simulations, based on the rigorous formalism of molecular physics, constitute possibly the most accurate and accessible approach to model protein motions with atomic detail. Although still an idealized description of reality—proteins diffusing in a crowded and complex cellular soup—MD is based on a careful parameterization of covalent and non-covalent forces on the atomic scale (Beauchamp et al., 2012; Lindorff-Larsen et al., 2012; Monticelli and Tieleman, 2013). Since the first eye blink 9.2 ps simulation of the small BPTI (McCammon et al., 1977), MD has evolved dramatically over the past 40 years up to become almost a “computational microscope” (Dror et al., 2012): it is expected that for relatively small systems like GPCRs, MD will reach the second scale within 5 years (Martínez-Rosell et al., 2017). Nevertheless, for average protein machines, transitions are difficult to sample due to inherent stochasticity and high-energy barriers, involving challenging time and length scales. Although specialized computers like Anton allow simulations of ever-larger systems, longer than ever, and have indeed brought novel insights for key drug targets like GPCRs (Dror et al., 2015), Voltage-gated channels (Jensen et al., 2012) or Kinases (Shan et al., 2012, 2014), conformational changes are still hard to catch. As a rule of thumb, “everyday” simulations invariably require algorithmic “tricks” to explore transitions with reasonable efficiency. More than computational power or simulation length, efficient sampling remains a bottleneck.

The next brief enumeration of MD-strategies to overcome this problem and explore large transitions should provide the reader with a clear picture of its complexity and its many potential pitfalls. Without aiming to be exhaustive (for detailed reviews see e.g., Bernardi et al., 2015; Maximova et al., 2016; Mori et al., 2016; Harpole and Delemotte, 2018), the most common “tricks” (Pietrucci, 2017) to explore transitions are broadly: first, to speed up or optimize exploration of the FEL, without modifying it; second, to actually change the FEL to easily move and jump across its “hills and valleys” (Table 1). In both cases, the search can be biased or directly pushed along some *a priori* “direction,” i.e., a CV. Among the first group are many multi-replicate methods, well-suited for highly scalable software implementations thanks to their intrinsically parallel algorithms. Replica exchange MD (REMD) often called “parallel tempering” [first applied to MD in Sugita and Okamoto (1999), reviewed in Ostermeir and Zacharias (2013)], exchanges multiple trajectories run in parallel (typically at different temperatures) to escape local minima. Weighted ensemble methods (WEM) originally developed for simpler Brownian Dynamics (BD; Huber and Kim, 1996; see also Zuckerman and Chong, 2017), use quasi-independent trajectories in which individual runs spawn daughter trajectories upon reaching new “bins” of the configuration space. Mention apart deserves adaptive-MD, a general term which includes a wide array of multi-run schemes aimed to speed up rare events without explicit biasing (Bowman et al., 2010; Pronk et al., 2011; Doerr et al., 2016). The main idea behind adaptive-MD is that simulations are guided toward *underexplored* FEL regions via iterative *on-the-fly* analysis; similarly, WEM partition of the FEL into bins also needs previous CV-reduction. Therefore, to identify meaningful CVs to check how simulations proceed becomes central, with risks to generate overly smooth landscapes or distort transition mechanisms (see Hruska et al., 2018; Zimmerman et al., 2018). One analysis approach used to guide sampling in adaptive-MD, are Markov State Models (MSMs; Pande et al., 2010), a statistical method to describe dynamics as memory-less transitions between states. MSMs can infer long-timescale dynamics from sets of shorter simulations, providing yet another shortcut to the sampling conundrum (Chodera and Noé, 2014). In contrast to these costly multi-replicate schemes, biasing methods directly guide single simulations through relevant CVs. For example, Essential Dynamics (Amadei et al., 1993; Daidone and Amadei, 2012) extracts with PCA the “essential” CVs (Essential Modes), which are used to bias the sampling toward collective motions. In Dynamic importance sampling (DIMS; Zuckerman and Woolf, 2000; Perilla et al., 2011) a progress variable or CV is used to select the most productive movement toward the target in a MC-scheme, while in Temperature-Accelerated MD (TAMD; Maragliano and Vanden-Eijnden, 2006) temperature is increased specifically along selected CVs.

A completely different approach is taken in FEL-modifying approaches like Umbrella sampling (US; Torrie and Valleau, 1977), which introduces harmonic biasing potentials along CVs in overlapping “umbrella” windows. Accelerated MD methods (aMD; Hamelberg et al., 2004; Pierce et al., 2012) change the relative height of the basins by adding “boost” potentials when the system's energy falls, locally flattening the FEL. In metadynamics (MTD), free energy wells are filled with “computational sand” to prevent returning back to previously explored CV-regions (Laio and Parrinello, 2002; Laio and Gervasio, 2008). The accelerated weight histogram (AWH; Lindahl et al., 2014, 2018) adaptively bias simulations to fit a target distribution, filling up energy minima in a similar spirit as MTD (see Figure 1C), while in conformational flooding (Grubmüller, 1995), a destabilizing potential is added to the starting state, lowering the transition barrier. From all above methods, MTD has been maybe the most widely applied to study large transitions in a number of pioneering works, from the opening/closing of kinases (Berteotti et al., 2009) or actin monomers (Pfaendtner et al., 2009) to flexible binding and dissociation events (Limongelli et al., 2010, 2012; Formoso et al., 2015).

Moreover, all these different approaches can be combined in virtually infinite ways, giving rise to hybrid methods like Bias-Exchange MTD (Piana and Laio, 2007), MSM-driven MTD (Sultan and Pande, 2017), and many others. The main shared concern for the above listed methodologies is that trajectories may not accurately reproduce the biologically relevant motions (i.e., trapped experimentally), since they either modify the way sampling is done by decreasing its randomness, or directly change the underlying landscape, which can require re-scaling to remove biasing. Particularly, the bias-introducing methods require extra caution to not produce unrealistic high-energy intermediates (Ma and Karplus, 2002; Ovchinnikov and Karplus, 2012). A tightly connected issue stems from the choice of CVs, which is critical (Pan et al., 2014) but nevertheless, is frequently defined *ad-hoc* for each system. Typically, CVs are defined in terms of e.g., radius of gyration, distances, angles, rMSDs changing across sets of trial trajectories, which are expected to correlate or “describe” the transition. MSMs (Sultan and Pande, 2017), or machine-learning (Chen et al., 2018) can also be applied to solve this “dimensionality reduction” problem and identify relevant CVs. Another alternative is to define CVs from experimental data e.g., NMR chemical shifts (Granata et al., 2013) or SAXS intensities (Kimanius et al., 2015). In summary, CV definition is a non-trivial problem. For all these reasons, unbiased long simulations, which neither perturb the FEL nor require previous CV knowledge, are often preferred alternatives in many studies aiming for experimental validation, as we will review in the last section.

## Path-Finding Methods: Throwing Ropes Over Mountains

Apart from the host of methods to enhance MD conformational sampling, there is another fundamental strategy to explore protein transitions: to simplify either the simulation algorithm or the system, in order to obtain just a feasible pathway between states. Finding transition paths has been compared to “*throwing ropes over mountain passes in the dark”* (Bolhuis et al., 2002; Dellago and Bolhuis, 2007), since indeed, such methods produce one-dimensional trajectories, like ropes in the conformation space (Figure 1C). Instead of sampling transition ensembles covering broad areas of the FEL, the goal of path-sampling methods is to generate sequences of structures connecting end-states. Such rope-like transitions, apart of providing first mechanistic insights, can serve as seeds for further MD (e.g., with US, MTD or “swarms-of-trajectories” Pan et al., 2008; Maragliano et al., 2014) to reconstruct the FEL.

Very broadly, path-generating methods (Weiss and Koehl, 2014; Table 1) can be also classified into two groups: (i) geometric morphing algorithms, which generate stereochemically correct morphs between structures, without any potential function, and (ii) those methods based on some potential energy, that actually attempt to approach minimum energy paths (MEPs) connecting basins. Among the latter, there are path-finding schemes based on MD inspired by the same ideas of enhanced sampling, along with a series of CG-methods, which take a entirely different approach, simplifying description of structures and their interactions.

The first online tool to compute transition pathways appeared within the MolMov Database (MolMovDB; Gerstein and Krebs, 1998; Krebs and Gerstein, 2000), and applied the simplest possible morphing: a linear interpolation in Cartesian coordinates, followed by energy minimization. As could be expected, MolMovDB paths project as perfectly straight lines in the experimental PC-landscape, and thus do not correspond at all to realistic transitions (Figure 1C, left). FATCAT also uses a interpolation of rigid-body motions (Ye and Godzik, 2004). More sophisticated are methods like FRODA (Wells et al., 2005) or geometric targeting (Farrell et al., 2010), which move atoms toward the target by enforcing geometric constraints to keep stereochemistry, while robot motion-planning algorithms (Cortés et al., 2005; Haspel et al., 2010; Al-Bluwi et al., 2012) exploit analogies between molecular bonds and robot links to perform fast molecular kinematics. Note that none of these geometric path-finding methods, which usually generate atomistic paths thanks to high computational efficiency, aims to provide a physical approximation to the FEL. This is not the case for MD-derived perturbation methods (Huang et al., 2009) like targeted (Schlitter et al., 1994), steered (Izrailev et al., 1997), or adiabatic MD (Marchi and Ballone, 1999; Paci and Karplus, 1999), where an MD simulation is directly pushed to the target by time-dependent potentials along a CV. In the so-called “*chain-of-states”* methods (Tao et al., 2012) like the nudged elastic band (Maragakis et al., 2002) or the string methods (Ren and Vanden-Eijnden, 2005; Ren et al., 2005; Ovchinnikov et al., 2011), serial images of the system are minimized to find MEPs; in the “path-method,” a guess path coordinate and two CVs that are functions of it are introduced to locally explore and optimize pathways (Branduardi et al., 2007; Bonomi et al., 2008). Although all these enhanced MD-derived path- sampling methods can be faster than conventional MD, finding proper CVs, biasing definitions or initial paths is again critical, and thus their implementation is not straightforward.

In contrast with the MD-inspired methods, CG-approaches, more than as alternative methods, should be rather considered a different way of looking at the sampling problem, literally, from a more collective scale. Coarse-graining simplifies the description of a system to capture its behavior with a minimum of parameters (Tozzini, 2010; Orozco et al., 2011; Saunders and Voth, 2013). By simplifying both potentials and structure description (Kmiecik et al., 2016), CG-methods accelerate computation increasing orders of magnitude the accessible scales; metaphorically speaking, they would be analogs to approaches like cryo-EM or SAXS, in which detail can be sacrificed to gain information from very large or flexible systems. Although hampered by loss-of-resolution regarding time and chemical properties, CG-methods can thus provide deep insights into complex systems behavior, as they distill multidimensional information to its very essential features. Although there are CG-force fields like the popular MARTINI implemented into real MD schemes (Marrink and Tieleman, 2013; Ingólfsson et al., 2014), in general CG-models are used in the context of much simpler algorithms, typically produce one-dimensional pathways, and are often available as webservers (Table 1).

To generate quick and efficient transitions, CG path-finding methods (Zheng and Wen, 2017) use a host of conceptually diverse protein representations: from a few heavy atoms (e.g., CABS model; Jamroz et al., 2013; Kmiecik et al., 2016) to residue beads (typical of ENMs) or rigid domains; and the same holds true for algorithmic approaches, that span from matrix diagonalization to MC or BD simulations. The only thing they have in common is skipping MD computational limitations, at the cost of losing information about time and energy. Among CG-methods, ENMs (Chennubhotla et al., 2005; Bahar et al., 2010) stand out due to conceptual simplicity and power to predict large changes through Normal Mode Analysis (NMA; Case, 1994). NMA is a molecular mechanics technique based on harmonic potentials, which was first used to predict infrared spectra and soon became also applied to analytically compute near-equilibrium protein atomic oscillations (Brooks, 1983; Levitt et al., 1985): solving a simple eigenvalue problem, a vector describing the directions of movement for every atom could be obtained. Inspired by “*beads-and-springs”* polymer models (Flory et al., 1976; Go and Scheraga, 1976), further coarse-graining of the protein description up to the C-alpha backbone lead to the minimalist ENM-NMA (Tirion, 1996; Bahar et al., 1997; Atilgan et al., 2001). Typically, ENMs reduce protein architecture to a network of Cα-carbons connected by springs, which model covalent and non-covalent interactions. In spite of this simplicity, it soon became evident that ENMs can not only predict residue fluctuations, but are also capable of guessing with striking precision the directions of large-scale transitions between e.g., X-ray open and closed pairs (Tama and Sanejouand, 2001). Later work has shown that ENMs reproduce as well the flexibility from experimental X-ray and NMR ensembles, or long MD simulations (Rueda et al., 2007; Orellana et al., 2010; Mahajan and Sanejouand, 2015; Sankar et al., 2018) and importantly, track the pathways for conformational change (Orellana et al., 2016; see NM projections, Figure 1C, left). Therefore, ENMs have been at the core of CG-strategies to find transition paths; however, being limited to an equilibrium basin, pathway generation requires iterative deformation along selected NMs, or implementation into some simulation scheme. Iterative ENMs range from simple interpolations like NOMAD-Ref and others (Kim et al., 2002; Lindahl et al., 2006; Seo and Kim, 2012) to more complex two-state approaches like iENM or ANMPathway (Yang et al., 2009; Tekpinar and Zheng, 2010; Das et al., 2014) or MinActPath (Franklin et al., 2007; Chandrasekaran et al., 2016), which assumes harmonic potential at the end-states and solves the action minimization problem to find the crossing points. A common issue for such CG-methods is that they typically produce stereochemical distortions, which can be reduced using internal coordinates like in iMODS (López-Blanco et al., 2014), structure corrections in NMSIMs (Ahmed et al., 2011; Krüger et al., 2012), or conjugate peak refinement like in the plastic network model (PNM; Maragakis and Karplus, 2005). In general, these approaches share the ENM power to capture allosteric transitions, but also display a shared weakness: a trend to reproduce similar one-dimensional paths rather than random pathway ensembles (Figure 1C, center). One solution to this problem is using NMs to bias simple e.g., Discrete Dynamics (dMD) simulations (Sfriso et al., 2012, 2013) in order to obtain a wider ensemble, although still, mode selection, as CV selection in enhanced MD schemes, poses a problem. Recently, we proposed an ENM-driven simulation approach, eBDIMS (Orellana et al., 2016, 2019a), also performing in parallel a thorough validation of path-finding algorithms against multi-state ensemble PCA. Based on a refined ENM force-field (Orellana et al., 2010), eBDIMS generates paths driven by interresidue distances, using a DIMS-Langevin scheme with a friction term mimicking solvent. This avoids unrealistic deformations, at the same time that ENM-modes are spontaneously sampled, generating random and non-linear trajectories.

Mention apart deserve hybrid methods like Climber (Weiss and Levitt, 2009), which iteratively pulls the interresidue distances adding harmonic restraints to an internal energy function, based on the atomistic ENCAD atomistic force-field (Levitt et al., 1995). In our comparative studies we found that eBDIMS and Climber, starting from entirely different approaches (CG- vs. atomistic, Langevin integration vs. iterative pulling/minimization), generate surprisingly convergent, non-linear, and asymmetric paths in PC-space. Remarkably, these paths closely overlap with solved experimental intermediates, which delimit the areas typically sampled by MD (see Figure 1C, center and right). Overall, our findings strongly suggested that these non-linear path-finding methods converge to actual MEPs, which are populated by trapped experimental intermediates. This raises a important question: how is it possible that such simple C-alpha based harmonic models like eBDIMS, can predict the directions of non-equilibrium conformational changes, while MD often requires powerful computing or enhanced sampling? On one hand, it has been suggested that dynamical systems theory assures the conservation of quasi-periodic motions upon small perturbations (Bastolla, 2014), and thus, ENMs are valid beyond the equilibrium, and in a wider set of conditions than was previously thought. On the other hand, the evident power of CG-methods to predict large-scale transitions and intermediates trapped by cryo-EM and crystallography, not only demonstrates such validity, but more importantly, it confirms that the collective shape-encoded dynamics of proteins, is maybe an essential determinant driving their underlying biologically functional transitions. Therefore, CG-methods are not just a quicker alternative to MD, but can provide an essential tool to dissect multi-scale problems like protein large transitions (Voth, 2009), specially in schemes where they are integrated with MD and experiments [see e.g., our recent experience (Orellana et al., 2019b) briefly discussed below].

## Cross-Validation of Simulations and Experiments: Toward Integrative Biology

Although the “*raison d'être”* of most theoretical methods to model protein transitions is to gain insight into molecular mechanisms and connect them to biology, attempts to validate them are still rare, and thus, any *in silico* predictions usually remain in the computational realm as mere hypotheses and are looked at with suspicion by experimentalists (see Lowe, 2015 critique on Kohlhoff et al., 2014). Traditionally, MD provided dynamic information on microscopic scales often inaccessible by experimental probes (e.g., atomic details on hydrogen bonding, loop fluctuations, etc.), and thus were un-testable. As larger scale events like conformational changes are simulated, MD can generate semi-quantitative estimates of observables that can be more easily measured experimentally. Therefore, current MD can significantly contribute to the understanding and interpretation of experimental data; and alternatively, it can also be driven by experiments (Hollingsworth and Dror, 2018). However, in comparison with the large efforts concentrated on pushing the simulation length and sampling, little has been done to systematize and validate in parallel the information obtained, especially when approaching the scales in which transitions happen and propagate over.

Simulating the physical world always involves a degree of approximation and uncertainty (Berendsen, 2007); but the same is true for biological experiments. This constitutes maybe the core reason separating the *in silico* world from actual biology: the extraordinary difficulty posed by integration of atomic-level data on motion with higher-scale experiments, which typically average out dynamic properties over time and space. Recently, a thorough critical analysis of factors influencing the agreement of simulations and experimental data was presented by van Gunsteren et al. (2018). We will not discuss here related issues associated to force-field parameterization (Lindorff-Larsen et al., 2012), convergence of the simulations (Knapp et al., 2011; Sawle and Ghosh, 2016), prediction of microscopic observables (Childers and Daggett, 2018), or the multiple caveats of modeling more realistic e.g., crowded complex environments (Chavent et al., 2016), electrochemical gradients (Delemotte et al., 2008; Khalili-Araghi et al., 2013), etc. We aim rather to revisit some experimental approaches that have recently provided *hands-on* direct or indirect validation of *in silico* predicted large-scale transitions (Table 2).

While there has been extensive work on force-field parameterization e.g., benchmarking predictions about microscopic properties, studies benchmarking the performance of atomistic simulation methods to sample conformational transitions are limited and often reduced to small proteins (Pan et al., 2016). A related issue with MD- benchmarking is also the abovementioned difficulty to identify relevant CVs for complex systems, especially when only one of the conformational states is known. Note that, in contrast to MD, benchmarking against large-scale changes not only has routinely been done for CG-methods, but also constituted the main basis for their parameterization and in consequence, are extremely effective at predicting transitions along with their CVs. Independent of the strengths and weaknesses of each method, however, the main issues to validate transition pathways are essentially two: on one hand, the scarcity of experimental data about on-pathway intermediates; on the other, the uncertainty determining the relevant CVs to monitor changes and their associated observables. Although a transition pathway should be ideally supported by direct structural data (either crystallography, cryo-EM, NMR, or SAXS), this is often difficult and the only feasible option is to attempt indirect “soft” validation, either from distance parameters e.g., via single-molecule FRET, FACS, or from functional assays, which can test predictions about protein activity, as we briefly review next.

## Direct Path-Validation: Protein Data Bank Ensembles and Landscapes

Classically, *in silico* pathways like those generated by path-sampling were evaluated on the sole basis of stereochemical quality, or by tracking progression along *ad-hoc* system-defined coordinates (Das et al., 2014; Seyler and Beckstein, 2014). As mentioned above, the selection of heuristic CVs for dimensionality reduction is problematic (Seyler et al., 2015), and in practice, structural quality or progression along user-defined CVs does not assure that a pathway samples the biologically relevant routes. Weiss and Levitt clearly stated this question a decade ago: “*Can morphing methods predict intermediate structures?”* (Weiss and Levitt, 2009), proposing for the first time to benchmark against proteins with at least three distinct states solved, and asses how close sampled pathways spontaneously approach known intermediates in terms of rMSD. Although this procedure definitely poses a more accurate test for *in silico* pathways, it cannot assess the feasibility of the movements or to what extent they correspond to biological motions. Based on these ideas, we proposed to go beyond two- or three-state benchmarking by introducing ensemble-level analyses that consider all structural information available in the PDB for a given protein, extracting at the same time their intrinsic CVs using PCA (Orellana et al., 2016). This kind of validation provides an extremely stringent test to evaluate sampling both by MD and path-finding algorithms and, thanks to the increasing amount of multi-state structural data available, we foresee that it could become widely applicable in the near future with cryo-EM. As a case apart of “hard” pathway validation, it is necessary to mention the study on a SWEET transporter by Latorraca et al. (2017), in which the spontaneous transition toward the inward-open state was first observed *in silico* with Anton simulations, and subsequently validated by determining an X-ray structure trapped in the same conformation. Although such an approach is not feasible to routinely validate pathways, it has provided maybe the strongest evidence to date in favor of the power of MD simulations to accurately sample the conformational space of proteins.

## Soft Validation: From FRET and Antibody Binding to Functional Assays and Animal Models

MD simulations have been traditionally validated and compared with microscopic information on relatively local protein flexibility like NMR couplings, B-Factors, etc. During the last years however, simulations have started to generate predictions of a scale that is suitable for experimental validation through non-structural techniques, finally crossing the boundaries with molecular biology. A quick glimpse into recent examples of cross-validation of conformational changes between simulation and experiments (Table 2) clearly shows how finally, we are starting to break the barriers separating both, providing new insights into biomedically relevant systems, including key drug targets. Functional conformational changes usually involve either large rigid-body motions of structural elements or more local unfolding, loop fluctuation transitions. While the first can expose or bury molecular surfaces for dimerization, interaction with other proteins or ligands etc., the second may have more subtle effects on structure-function relationship e.g., at enzyme active sites. Observing such changes *in silico*, has given rise to quantitative or qualitative predictions that mainly fall into two categories: concerning interactions with other proteins or small molecules (dimerization, binding), and/or regarding activity—catalysis, phosphorylation, ion transport, etc.

Maybe one of the first examples attempting soft validation of *in silico* transitions aroused from short simulations of open/closed changes in Hsp70, confirmed by Trp-fluorescence changes upon ATP binding (Woo et al., 2009). A more complex validation strategy was taken by Laine et al. (2010), designing and testing a series of inhibitors against the different conformations of an allosteric site throughout an *in silico* transition path. In a groundbreaking study of the EGF-Receptor (EGFR kinase domain; Shan et al., 2012), Anton simulations revealed a third intermediate state characterized by local αC-helix disorder; further simulations of mutations indicated that they suppress this disorder to enhance dimerization and activation. In this case, proving intrinsic disorder and mutation effects required Hydrogen/Deuterium (H/D) exchange mass spectrometry (Wales and Engen, 2006), while enhanced dimerization was shown by Blue Native Gel electrophoresis (Wittig et al., 2006). Later work by the Shaw group, cross-validating NMR data and simulations (Arkhipov et al., 2013; Endres et al., 2013), provided new insights into EGFR transmembrane dimerization. Shorter μs-simulations by Kaszuba et al. (2015) also led to predictions about the impact of glycans on EGFR conformation, which were tested by monitoring the accessibility of glycosylation-sensitive surface-epitopes. Recently, we combined first a mutational screening partly based on ENMs, followed by MD simulations of “dynamically” hot EGFR ectodomain mutations (Orellana et al., 2014, 2019b) in a multiscale CG-MD scheme similar to that proposed by Saunders and Voth (2013). This approach highlighted how, as happens often experimentally, mutagenesis can help to trap intermediate states. In this case, the MD-trapped transition state, happened to be the target for a therapeutically relevant antibody, mAb806, which had been long hypothesized to bind a third ectodomain conformer distinct from the known crystal structures and enriched in tumor cells. This provided a rare opportunity to directly extrapolate an MD prediction to animal models by testing mAb806 therapeutic impact, with surprising success (Binder et al., 2018; Orellana, 2019); moreover, the integration of functional experiments, SAXS and MD revealed unsuspected functional and allosteric convergence of ectodomain deletions and missense mutations. A similar example, in which a protein is known to perform a certain biological activity but the corresponding conformation remains elusive, is illustrated by the work by Machtens et al. (2015), which extended previous MTD findings by Grazioso et al. (2012). In this case, excitatory aminoacid transporters (EAATs) were known to transport anions but the specific conduction path was not obvious in end-state X-ray structures. ED simulations of a prokaryotic glutamate transporter homolog, Glt_ph_, revealed a potential channel in an intermediate state (independently trapped with crystallography), and the predicted pore-lining residues were confirmed with Trp-scanning mutagenesis, fluorescence quenching, and electrophysiology. Another indirect approach to validate MD-predicted changes consists on assessing intra or intermolecular distances with FRET, used e.g., to confirm the compaction of importin in apolar solvents (Halder et al., 2015) or DEER, an approach that allowed to prove the opening/closing dynamics in heterotrimeric G-proteins (Dror et al., 2015) and its modulation by nucleotide binding.

Although not the subject of this review, it is worth to mention the advances on simulations of spontaneous ligand binding events and protein-protein interactions, which constitute a special case regarding experimental validation and can occasionally provide indirect validation for conformational changes related to binding. For example, either long simulations or enhanced sampling techniques like aMD or MTD have captured spontaneous binding of small molecules to protein kinases or GPCRs (Dror et al., 2011, 2013; Shan et al., 2011, 2012; Kappel et al., 2015), dimerization in membranes (Lelimousin et al., 2016), or protein-protein interactions (Ma et al., 2019), approaching or reproducing crystallographic binding poses or NMR ensembles. In these cases, the PDB coordinates of known complexes, together with free energies of binding, drug efficacies, etc. (Shukla et al., 2015) can provide a hard-validation for MD.

## Concluding Remarks

We have provided a brief overview of the multiple approaches that are used to explore the conformational landscapes of proteins and their transitions *in silico*, and reviewed different methods used for their validation. On one hand, it becomes clear that the accumulated structural information and flexibility-capturing techniques like cryo-EM are revealing first glimpses on functional landscapes. On the other hand, computational methods have reached maturity and are entering a stage in which they can start to contribute to real biology, modeling longer and larger scales. We have revisited the many approaches available to explore the FEL of proteins, optimizing hardware, software and algorithms pursuing the dream of the seconds-long sampling. From a completely different standpoint, simulations in crowded cell-like soups of multiple copies of the same protein, although still in the ns-scale, are already a reality that holds promise to reveal dynamical complexity in local microenvironments, providing yet another approach to the sampling problem (Yu et al., 2016; Feig et al., 2018). We have also briefly mentioned machine learning algorithms, paradigmatic of a series of novel fast-developing non-physically based strategies which are gaining ground to study transitions, either alone or in combination with MD or CG-methods: from co-evolution analysis (Morcos et al., 2013; Sutto et al., 2015; Sfriso et al., 2016) to cross-correlation, network and community approaches (Potestio et al., 2009; Morra et al., 2012; Rivalta et al., 2012; Papaleo, 2015; Negre et al., 2018), neural networks and deep learning (Ung et al., 2018; Degiacomi, 2019), or integrative sequence and structural analysis (Flock et al., 2015). These approaches, not primarily intended to generate conformational pathways or obtain a physical FEL, have shown their power to reveal new alternative conformations and dissect allosteric mechanisms, and thus are also greatly contributing to the exploration of protein flexibility space. We have reviewed some of the many flavors of CG- models and algorithms, and how they can provide low-resolution but stunningly accurate pathways. Finally, we have discussed recent examples where simulations have trapped intermediate states before confirmation by X-ray crystallography (Latorraca et al., 2017), or by *in vivo* tumor models (Orellana et al., 2019b). Altogether, the explosion of structural data, along with the ever expanding toolkit of *in silico* methods, computer capabilities and growing integration between simulations and experiments—driving or being driven by them—are beginning to fulfill the dream of connecting the micro-, meso-, and macro- scales in the study of life phenomena. It also becomes evident that this enterprise requires careful integration of a multitude of techniques and approaches, to connect the atomistic level with the emerging collective behaviors that rule conformational changes. The times ahead are exciting, as we are approaching a critical mass of information on protein structures, and experimental techniques allow exploring their dynamics with ever-increasing detail. The challenge will be to merge the ever-growing data into a coherent picture, which has certainly the potential to revolutionize biology, medicine and drug discovery.

## Author Contributions

LO conceived and wrote the manuscript and prepared figures.

### Conflict of Interest

The author declares that the research was conducted in the absence of any commercial or financial relationships that could be construed as a potential conflict of interest.
